# Revealing Elasmobranch Distributions in Turbid Coastal Waters: Insights From Environmental DNA and Particle Tracking

**DOI:** 10.1002/ece3.70857

**Published:** 2025-01-24

**Authors:** Nick Dunn, Sophie Ward, Joanna Barker, Jake Davies, Sarah Davies, Ben Wray, Peter Robins, Isabelle Apetroaie, Jake Williams, Kevin Hopkins, David Curnick

**Affiliations:** ^1^ Institute of Zoology of the Zoological Society of London London UK; ^2^ School of Ocean Sciences Bangor University Menai Bridge Anglesey UK; ^3^ Conservation and Policy Zoological Society of London London UK; ^4^ Natural Resources Wales Bangor Maes y Ffynnon Bangor Wales UK; ^5^ Imperial College London—Silwood Park Campus Ascot UK; ^6^ University College London Research Department of Genetics Evolution and Environment London UK

**Keywords:** eDNA, hydrodynamic modelling, marine, metabarcoding, sharks and rays

## Abstract

Many sharks, rays and skates are highly threatened and vulnerable to overexploitation, as such reliable monitoring of elasmobranchs is key to effective management and conservation. The mobile and elusive nature of these species makes monitoring challenging, particularly in temperate waters with low visibility. Environmental DNA (eDNA) methods present an opportunity to study these species in the absence of visual identification or invasive techniques. However, eDNA data alone can be difficult to interpret for species monitoring, particularly in a marine setting where its distribution can be influenced by water currents. In this study, we investigated the spatial and temporal distribution of elasmobranch species in two Special Areas for Conservation (SAC) off the coast of Wales. We took monthly eDNA samples for 1 year (starting September 2020 and March 2022 for the northern and southern SACs, respectively), and used metabarcoding to reveal the presence of elasmobranch species. We combined these data with hydrodynamic modelling and particle tracking methods to simulate the potential origins of the detected eDNA. We detected 11 elasmobranch species, including the critically endangered angelshark ( *Squatina squatina* ) and tope ( *Galeorhinus galeus* ). Most detections were in the spring and the fewest in the autumn. The particle tracking simulations predicted that eDNA was shed, on average, approximately 7 km and 15 km (in the northern and southern SACs, respectively) from the sampling stations at which it was detected. These results show that the two SACs represent important areas for elasmobranchs in the United Kingdom and demonstrate that eDNA methods combined with particle tracking simulations can represent a new frontier for monitoring marine species.

## WELSH LANGUAGE ABSTRACT

Mae llawer o siarcod a morgathod dan fygythiad difrifol ac yn agored i or‐ecsbloetio, felly mae gwaith dibynadwy i fonitro elasmobranciaid yn allweddol i reolaeth a chadwraeth effeithiol. Mae natur symudol a dirgel y rhywogaethau hyn yn ei gwneud hi'n anodd eu monitro, yn enwedig mewn dyfroedd tymherus â gwelededd isel. Mae dulliau DNA amgylcheddol (eDNA) yn rhoi cyfle i astudio'r rhywogaethau hyn yn absenoldeb y gallu i'w hadnabod yn weledol neu drwy ddefnyddio technegau ymyrrol. Fodd bynnag, gall data eDNA yn unig fod yn anodd ei ddehongli ar gyfer monitro rhywogaethau, yn enwedig mewn lleoliad morol lle gall cerrynt dŵr ddylanwadu ar ei ddosbarthiad. Yn yr. astudiaeth hon, fe wnaethom ymchwilio i ddosbarthiad gofodol ac amserol rhywogaethau elasmobranciaid mewn dwy Ardal Cadwraeth Arbennig (ACA) oddi ar arfordir Cymru. Fe wnaethom gymryd samplau eDNA misol dros gyfnod o flwyddyn (yn dechrau ym mis Medi 2020 a mis Mawrth 2022 ar gyfer yr. ACAau gogleddol a deheuol, yn y drefn honno), a defnyddio meta‐godau bar i ddatgelu pa rywogaethau elasmobranciaid a oedd yn bresennol. Cyfunwyd y data yma â modelu hydrodynamig a dulliau olrhain gronynnau i efelychu tarddiad posibl yr. eDNA a ganfuwyd. Canfuom 11 rhywogaeth o elasmobranciaid, gan gynnwys y maelgi ( *Squatina squatina* ) sydd Mewn Perygl Difrifol a'r ci glas ( *Galeorhinus galeus* ). Roedd y rhan fwyaf o'r darganfyddiadau yn y gwanwyn a'r lleiaf yn yr. hydref. Roedd yr. efelychiadau olrhain gronynnau yn rhagweld y byddai eDNA yn cael ei ollwng, ar gyfartaledd, tua 7 km a 15 km (yn yr. ACA gogleddol a deheuol, yn y drefn honno) o'r gorsafoedd samplu lle cafodd ei ganfod. Mae'r canlyniadau hyn yn dangos bod y ddwy ACA yn cynrychioli ardaloedd pwysig ar gyfer elasmobranchs yn y Deyrnas Unedig ac yn dangos y gall dulliau eDNA ynghyd ag efelychiadau olrhain gronynnau gynrychioli ffin newydd ar gyfer monitro rhywogaethau morol.

## 1 Introduction

Elasmobranchs (sharks, skates and rays) are one of the most highly threatened taxonomic groups globally (Stein et al. 2018) with over one third of species thought to be threatened with extinction (Dulvy et al. 2021). Around the United Kingdom (UK), Elasmobranchs are commonly caught in mixed‐species fisheries and represent a large proportion of discards at‐sea (Silva and Ellis 2019). The majority of data on populations of elasmobranch species in UK waters comes from scientific trawl surveys (Chevolot et al. 2006; Ellis et al. 2004) and historical fishing records (Barker et al. 2022; Hiddink et al. 2019), with evidence that many populations have suffered severe declines (Ellis et al. 2005). Targeted fishing for several elasmobranch species is banned under UK law and certain species are managed through Total Allowable Catches (TACs) and the Wildlife and Countryside Act (Defra Shark, Skate and Ray Conservation Plan, 2011). However, there remains a lack of data on the current status of elasmobranch populations in UK waters.

Estuarine and coastal waters around the UK are predominantly turbid (Wilson and Heath 2019), whereby riverine suspended sediment loads (Worrall, Burt and Howden 2013) are well‐mixed through macro‐ or hyper‐tidal regimes and/or by wave action (Neill and Hashemi 2013). Because of this poor underwater visibility, the effectiveness of established non‐invasive methods for monitoring elasmobranchs, such as Baited Remote Underwater Video Systems (BRUVS) (Juhel et al. 2018; MacNeil et al. 2020) and underwater visual census (UVC) (Graham, Spalding and Sheppard 2010; Juhel et al. 2018), are limited. Recently‐developed environmental DNA (eDNA) methods have the potential to reveal spatial and temporal distributions of elasmobranchs (Simpfendorfer et al. 2016). Defined as genetic material shed into the environment by an organism, eDNA can originate from blood, tissue, scales, cells, mucus and excretions (Taberlet et al. 2018). Detection of eDNA does not rely on visual identification of target species, therefore methods are well suited to the detection of highly‐mobile, elusive marine species, such as elasmobranchs and for detecting species within turbid waters (Ip et al. 2021; Liu et al. 2022). Environmental DNA methods have been used to detect threatened elasmobranch species in turbid waters of the English Channel (Liu et al. 2022) and Singapore, revealing 47 elasmobranch taxonomic units, 16 of which were assigned to species level (Ip et al. 2021). Furthermore, eDNA metabarcoding methods have been used to reveal how elasmobranch communities change with geographical differences in levels of anthropogenic pressure and conservation effort in New Caledonia (Bakker et al. 2017) and in response to illegal fishing pressure in the Chagos Archipelago Marine Protected Area (Dunn et al. 2023). Consequently, eDNA methods are becoming an invaluable tool for filling data gaps, and for helping understand the diversity and distribution of elasmobranchs.

Once shed from the individual, eDNA can persist in the water column, whereby it can be transported by ocean currents many kilometres away from where it was released into the environment (Andruszkiewicz et al. 2019), raising questions about the relevance of eDNA methods for identifying fine‐scale species distributions (Deiner and Altermatt 2014; Jerde et al. 2016). However, there is a growing body of research that indicates eDNA methods can produce high resolution biodiversity data at local scales (Jeunen et al. 2019; West et al. 2020; Wilms et al. 2022). Environmental DNA is known to degrade exponentially over time (Harrison, Sunday and Rogers 2019), with many studies attempting to assess the rate of decay (e.g. Andruszkiewicz Allan et al. 2021; Jo et al. 2020; Sansom and Sassoubre 2017) given that the rate of decay will determine the length of time eDNA is detectable in a region. Water turbidity and the presence of suspended particles has been shown to impact the persistence of and detectability of eDNA (Stoeckle et al. 2017; Brandão‐Dias et al. 2023) likely through adsorption offering some stabilisation to the DNA molecules which delays breakdown. Estimates of general eDNA persistence in UK waters suggests that it has a half‐life of ~24 h and remains detectable for around 48 (Collins et al. 2018).

Attempting to understand the transport of eDNA in the marine environment is a relatively new area of research but follows on from studies investigating the transport of larvae in the ocean using numerical particle tracking models (Ward et al. 2023; Coscia et al. 2020; Robins et al. 2013). Particle tracking methods have been used to simulate the source of eDNA within water samples, thus enabling the prediction of species' location for fish (Andruszkiewicz et al. 2019) and elasmobranch (Jenrette et al. 2023) species. Some experiments that have investigated eDNA transport distance in water have found that invertebrate eDNA in freshwater could be detected up to 12 km from an upstream source (Deiner and Altermatt 2014), whereas Murakami et al. (2019) reported that fish eDNA was mostly detectable within 30 m of a discrete source in a marine setting.

Pen Llŷn a'r Sarnau (PLAS) Special Area for Conservation (SAC) and Carmarthen Bay and Estuaries (CBAE) SAC, are two marine protected areas along the Welsh coastline that contain several habitat features that are important for elasmobranchs, including shallow reefs and estuaries. Several species of elasmobranch are known to reside in the SACs, including the Critically Endangered angelshark *Squatina squatina* (Barker et al. 2022). However, elasmobranch communities along the Welsh coast and within these SACs remain poorly understood due to the complexities of monitoring elasmobranchs in turbid habitats. In this study, we investigated the effectiveness of eDNA metabarcoding techniques to assess the spatial and temporal distribution of elasmobranch species across the two SACs. Further, we developed high‐resolution coastal ocean models and Lagrangian particle backtracking models for each bay (and surrounding area) to estimate the potential dispersal pathways from where the eDNA was shed to where it was sampled, and to simulate potential connectivity between sampling stations (within realistic eDNA decay timescales), allowing us to determine the independence of our sampling stations in terms of water flow. This work highlights the potential of eDNA studies to better understand temperate and turbid habitats and environments that are important for endangered species and advances the spatio‐temporal application of eDNA in the context of habitat feature based conservation.

## 2 Methods

### 2.1 Study Sites

**FIGURE 1:**
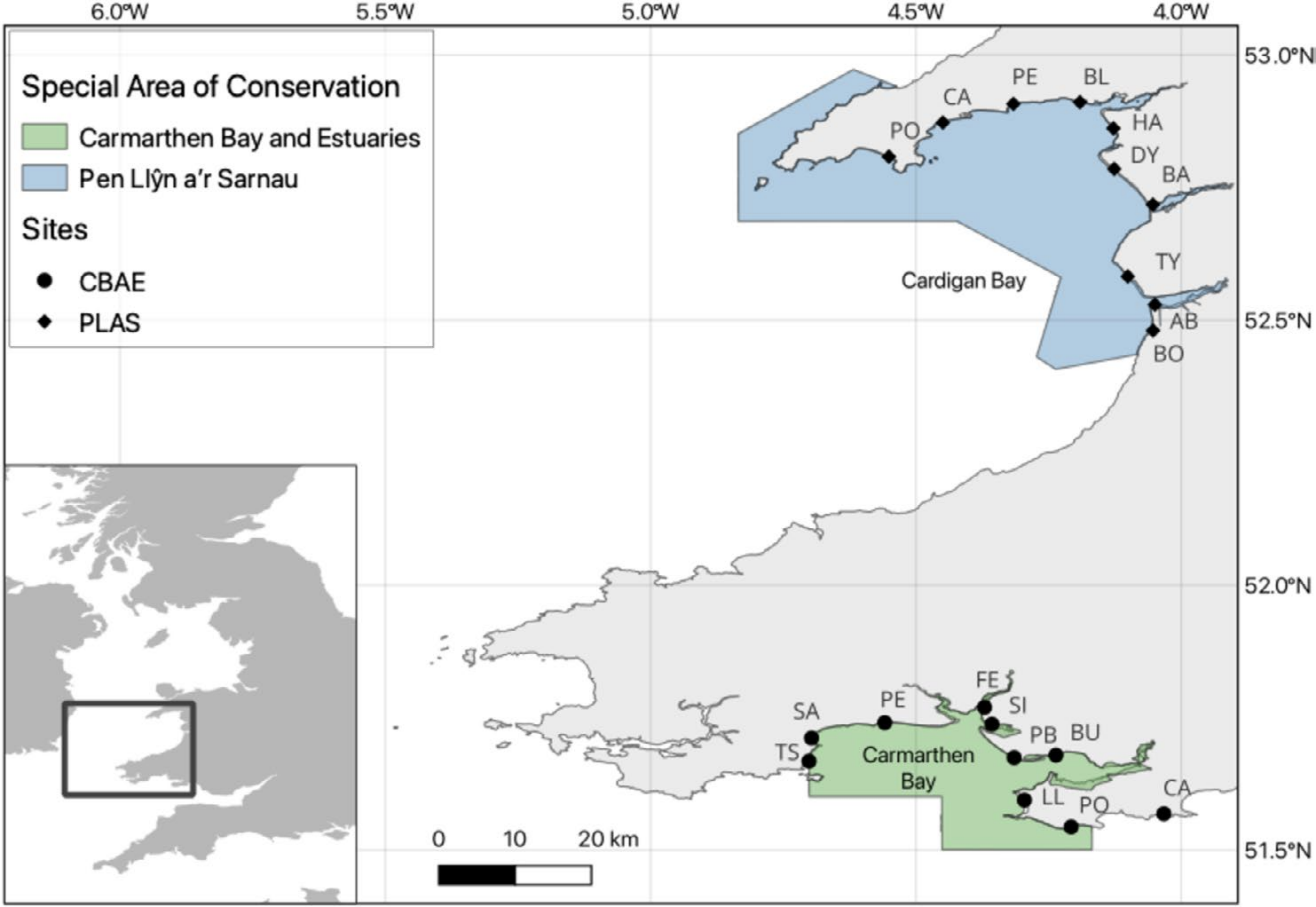
Sampling stations in the two Welsh Special Area for Conservation (SAC)s considered here: Carmarthen Bay and Estuaries (CBAE) (green area) and Pen Llŷn a'r Sarnau (PLAS) (blue area). The inset map in the bottom left illustrates the sites in the wider UK context. Each of the ten sampling stations in the two areas are indicated by black markers, and the abbreviated station names are given in full in the supporting information.

Water samples were taken monthly from ten stations within PLAS SAC, Wales, UK (September 2020–August 2021) and from ten stations within CBAE SAC (March 2022–February 2023) (Figure 1).

### 2.2 Sample Collection

At each sampling station, samples were collected by wading from the shore to waist‐depth (approx. 80 cm). Three 2 L collection bags were rinsed in the seawater and then used to collect 6 L of seawater. The collected water was then transported on ice to a field laboratory and filtered in three 2 L subsamples through 0.45 μm Whatman filters using vacuum filtration. If a filter became clogged during the 2 L, it was swapped for a fresh one and both were retained for analysis. Filters were folded and placed in individual 2 mL Eppendorf tubes and filled with Longmire's buffer for preservation. All filtration equipment was cleaned between samples (not between subsamples) by submerging in a 20% bleach solution for 30 min followed by submerging in deionised water for another 30 min to remove any bleach residue. After each clean, a negative control sample of 500 mL deionised water was filtered to track any potential contamination between samples, resulting in one negative control for every actual sample. The preserved filters were then transported to the molecular lab and stored at room temperature before DNA extraction, which took place within 3 months of sample collection.

### 2.3 DNA Extraction

DNA was extracted in a dedicated DNA extraction lab from each filter following the Spens et al. (2017) protocol for filters prepared with Longmire's buffer and using the Qiagen Blood and Tissue Kit (Qiagen). When multiple filters were used per sample, the lysis solutions after incubation of each individual filter were combined through the same spin column, resulting in one DNA extraction per station sample. DNA was eluted from the spin columns with 100 μL PCR‐grade water and this step was repeated to maximise yield and result in 200 μL of extracted DNA. At this stage, 100 μL from each triplicate was pooled for analysis and the remaining half of each replicate was archived for long‐term storage at −20°C. One DNA extraction control consisting of 500 μL ATL buffer was included with each batch of 23 sample extractions. All DNA extracts were quantified using a Qubit 4,0 fluorometer (Invitrogen).

### 2.4 PCR Amplification

Amplification of elasmobranch eDNA followed the MiFish protocol (Miya et al. 2015) using the Elas02 primers (Taberlet et al. 2018). The extracted DNA was amplified from the pooled samples in triplicate on a 96‐well plate in 12.5 μL reactions consisting of the following; 6 μL KAPA HiFi Hotstart Master Mix (KAPA), 0.7 μL of each 10 μM primer, 2 μL PCR‐grade water and 3 μL of extracted DNA template. Reactions were run on a G‐Storm GS1 thermal cycler with an initial denaturation step at 95°C for 2 min followed by 40 cycles of 95°C for 30 s, 55°C for 15 s and 72°C for 15 s, and completed with 7 min at 72°C. Each plate included a non‐template control (NTC) where PCR‐grade water was used in place of the DNA template and all negative field controls were amplified alongside the actual samples. The amplified products were visualised on a 1.5% agarose gel using GelRed stain (GelRed) and then cleaned using AMPure XP beads (Beckman Coulter), following the manufacturer's protocol and eluted with PCR‐grade water. No issues with amplification inhibition were identified in the gels, with all samples producing strong amplification bands so no further inhibition testing was performed.

A second stage PCR was then run to add Illumina index sequences to the PCR products, using the Nextera set A and set B index kits, each product was tagged with unique Illumina tags in the following reaction; 12 μL KAPA HiFi Master Mix, 2.5 μL index primer, 5 μL PCR‐grade water and 3 μL PCR product. This was run on a thermal cycler with an initial denaturation at 95°C for 3 min followed by 8 cycles of 95°C for 30 s, 55°C for 30 s and 72°C for 30 s and completed with 5 min at 72°C. The products were visualised on a 1.5% agarose gel using GelRed stain and cleaned using AMPure XP beads, following the manufacturer's protocol and eluted with PCR‐grade water.

Samples were sequenced in 4‐month sample batches, resulting in six runs with 86 to 92 libraries per run. Libraries were initially pooled in equal concentrations of 1 μL each and the pooled library was checked for correct base‐pair length using an Agilent TapeStation 2200, following the manufacturer's protocol. The pooled library was then run on an Illumina Miseq using a MiSeq Reagent Nano Kit v2 (300‐cycles) as a quality control run. This resulted in identifying the relative contribution of each individually indexed sample in the pooled library. For the data run, each individual library was pooled at volumes calculated from their relative contribution in the quality control run, to result in equal concentrations being loaded onto the MiSeq. These were sequenced using a MiSeq v2 300‐cycles reagent kit v2 with a 5% PhiX spike in control.

### 2.5 Bioinformatics

Sequences were obtained as demultiplexed FASTQ files from the Illumina MiSeq Reporter software and run through a custom bioinformatic pipeline (Williams et al. 2024, scripts available here: J‐Cos/SimpleMetaPipeline: 2.0.2). Demultiplexed sequences were trimmed, denoised and merged using a DADA2 pipeline in R to produce amplicon sequence variants (ASVs) (Callahan et al. 2016). Forward and reverse reads were truncated to 125 bp to allow adequate overlap for merging and merged sequences between 150 and 190 bp were retained for analysis. Sequence variants were curated with LULU (Frøslev et al. 2017), the match rate was set at 97% and the minimum relative co‐occurrence was set to 0.95. Taxonomy was assigned using IDTaxa (Murali, Bhargava and Wright 2018) and a curated classifier that was produced from the Metafish library (Collins et al. 2021) with the assignment confidence threshold set at 40% (moderate) (Murali, Bhargava and Wright 2018; Quast et al. 2013). After the creation of a phyloseq object using the phyloseq package in R (McMurdie and Holmes 2013), any ASVs present in the negative control for a given sample were removed from that sample in R.

All non‐elasmobranch reads were removed from the dataset and only samples containing elasmobranch reads were retained for further analysis. To limit the impact of sequencing and assignment error, singletons (sequences with only 1 read in a sample) were also removed from the dataset (Maiello et al. 2024), any remaining taxa were classed as a positive detection for analysis. Any sequences not assigned to genus level and sequences identified as contaminants from an alternative lab project were removed from the dataset, these were exclusively Indian Ocean elasmobranch species. Any remaining sequences present in the negative control for a given sample were also removed from that sample. Plots were made using ‘ggplot2’ to visualise the detections of each species by site and month. Species accumulation curves across each SAC were made using the iNEXT ShinyApp programmed in R (https://chao.shinyapps.io/iNEXTOnline/, Chao et al. 2014, Chao, Ma and Hsieh 2016).

### 2.6 Hydrodynamic Modelling

Hydrodynamic modelling combined with particle backtracking was used to predict the potential elasmobranch eDNA shedding locations from each eDNA sample station. We applied an ocean model (TELEMAC Modelling System v8p2; [www.opentelemac.org]) to simulate the hydrodynamics of the two SACs and surrounding coastal waters, during the fieldwork campaigns. The TELEMAC‐2D (two‐dimensional) module was used, which outputs depth‐averaged simulated variables (e.g. current velocities). Since the waters are generally shallow and well‐mixed in these regions, and the eDNA assumed to be neutrally buoyant within the water, the depth‐averaged modelling approach is appropriate. The hydrodynamic model is based on an unstructured triangular computational mesh which facilitates increased model resolution in regions of interest, such as coastal and estuarine areas, with coarser resolution offshore to optimise computational efficiency (e.g. Ward et al. 2023).

The ocean model grid was set with 3–5 km spatial resolution at the boundaries of the wider Irish Sea domain, increasing to < 150 m spatial resolution within the first couple of kilometres of Cardigan‐ and Carmarthen Bays, and to < 50 m horizontal resolution along the coasts and within the estuaries. Bathymetric data were mapped onto the mesh, comprising data from EMODnet (~115 m spatial resolution, www.emodnet‐bathymetry.eu), onto which high resolution (2–4 m) multibeam data were mapped, from UK Hydrographic Office ADMIRALTY Marine Data Portal (datahub.admiralty.co.uk/portal/). Additional high resolution LIDAR data (lle.gov.wales/catalogue/item/LidarCompositeDataset/) and multibeam data (e.g. Dyfi Estuary, see Harrison et al. 2022) were also incorporated into the bathymetric grids.

The TELEMAC‐2D model was forced at the open boundaries using tidal elevation amplitudes and velocities of 30 harmonic constituents derived from TPXO9 (TOPEX/Poseidon) global tide data which was 1/30 × 1/30° resolution [www.tpxo.net/global/tpxo9‐atlas]. Spatially‐ and temporally‐varying wind fields were incorporated into the model using output from the ERA5 reanalysis product from ECMWF [www.ecmwf.int/en/forecasts/datasets/reanalysis‐datasets/era5]. The hourly ERA5 10 m surface winds were used, available at a spatial resolution of ~30 km (~0.28°). River flow data (obtained from National River Flow Archive and Natural Resources Wales) were used, for PLAS (Rheidiol, Dyfi, Dysynni, Mawddach and Glaslyn) and for CBAE (Loughor, Tywi, Gwili, Dewi Fawr and Taf) (Appendix Table A1).

The model was run for 13.5 months spanning each set of sampling dates and, following a 30‐day model spin‐up, outputs were saved at instantaneous 5‐min temporal resolution, to be used to drive the particle tracking model.

### 2.7 Particle Backtracking Modelling

Theoretical ‘particles’ were simulated to represent the potential movement of discrete ‘packets’ of eDNA via Lagrangian particle backtracking, i.e., particle trajectories were simulated backwards in time, from the eDNA sample stations backwards to potential species source locations, over a defined eDNA decay timescale. The simulated currents output by the TELMAC‐2D model were used (by reversing their direction) to advect the particles backwards in time within the model domain. The simulated particles were transported passively, i.e., driven by advection from the simulated currents only, with no additional sub‐grid‐scale diffusive mixing included since the mesh resolution and model time step were sufficiently small (Guihou et al. 2018; Mayorga‐Adame et al. 2022). The particle tracking model was based on that of Ward et al. (2023), which includes a link to the repository containing the background model code.

We simulated potential eDNA source locations by backtracking virtual particles to their origins based on their arrival at sample stations. Particles were released from each of the ten sample stations within the two SACs, totalling 20. For each station, 1000 virtual particles were released from a 200 m radius centred on the sample location, accounting for variability in sample location and model resolution. These particles were released at the exact time of each corresponding water sample collection. This particle release procedure was repeated for each station and sampling time, resulting in 12,000 particles released per station over the 12 monthly sampling campaigns. In this backward particle tracking (hereafter referred to simply as ‘backtracking’), the model was configured to have particles arrive at the sample station at the precise sampling time. To account for the predicted 48‐h detectability window of eDNA and to allow a conservative spatial estimate to be made, the hydrodynamic model outputs were reversed to simulate particles moving backwards over the 3 days prior to the water sampling, with positions recorded at 5‐min intervals throughout this period. This 3 days time limit was used as a proxy for particle degradation, which was not itself otherwise parameterised in the model.

The total dispersal (via backtracking, averaged over the 12 months of particles released) was plotted from each sample station and presented as density distributions (‘heatmaps’). These heatmaps were generated by dividing the model domain into a regular 1 × 1 km grid. At each 5‐min timestep, the number of particles within each 1 km^2^ grid cell was counted and this number converted to a percent of the total number of particles within the domain (by dividing by total number particles released × timesteps). Thus, the total sum of the distributions in the heatmap is 100% (i.e., all particle locations counted at all timesteps) and the figures are useful to illustrate potential particle locations and range of transport, potential connectivity between the sampling stations, as well as identifying where particles tend to congregate (i.e., the highest density areas in the heatmaps). The analysis of the particle tracking was done in MATALB (version 2020a), and analysis and plotting scripts are available from: https://zenodo.org/records/14391532.

## 3 Results

**FIGURE 2:**
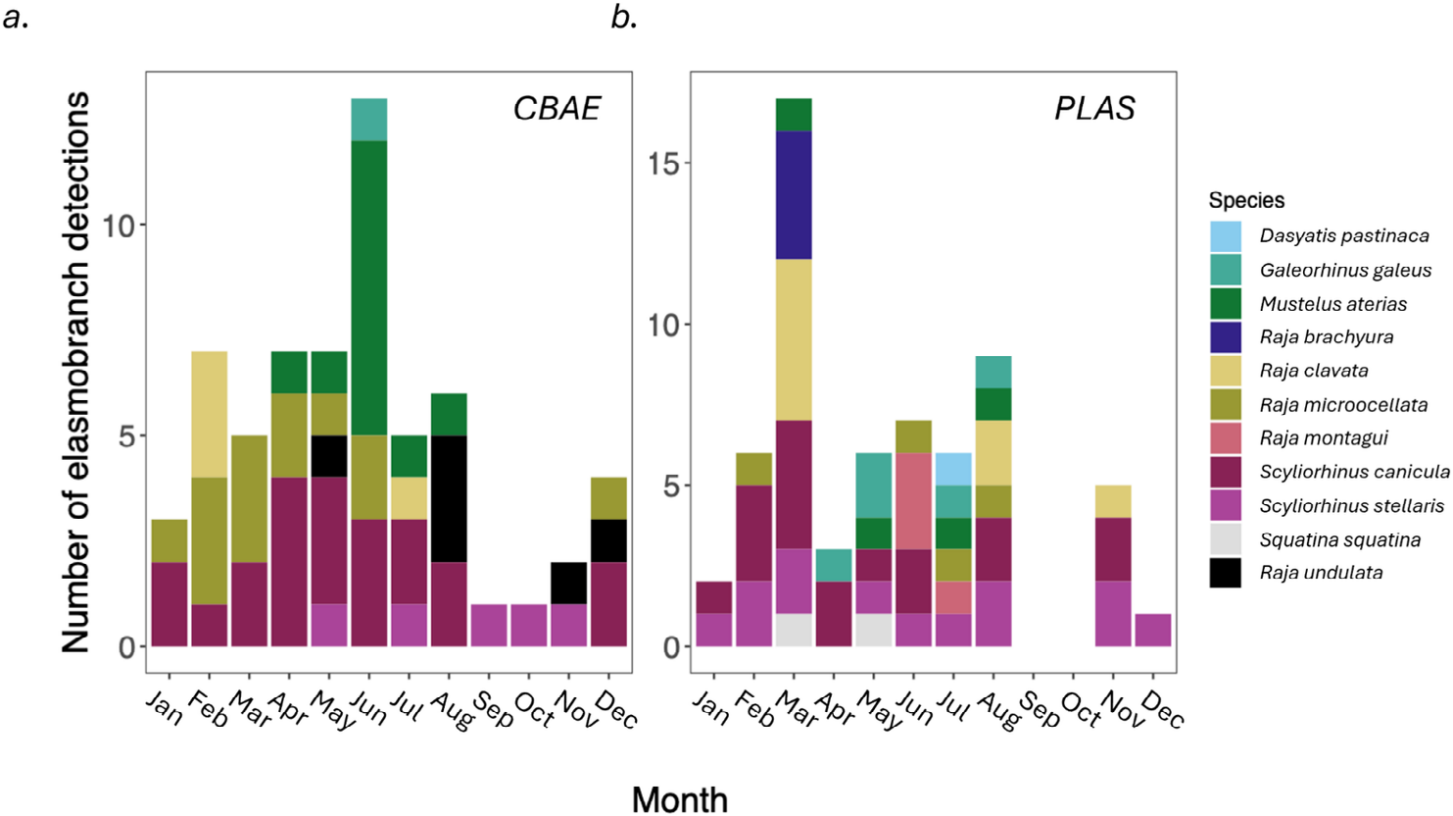
Elasmobranch detections by species and month across (a) CBAE SAC in 2022/23 and (b) PLAS SAC in 2020/21.

**FIGURE A1:**
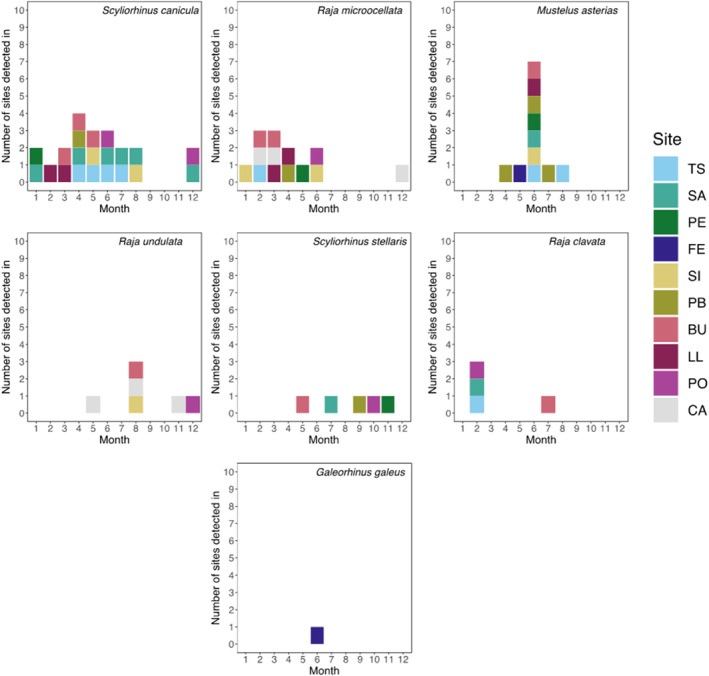
Number of detections for each elasmobranch species per month in CBAE SAC. Months are numbered according to the order January to December.

**FIGURE A2:**
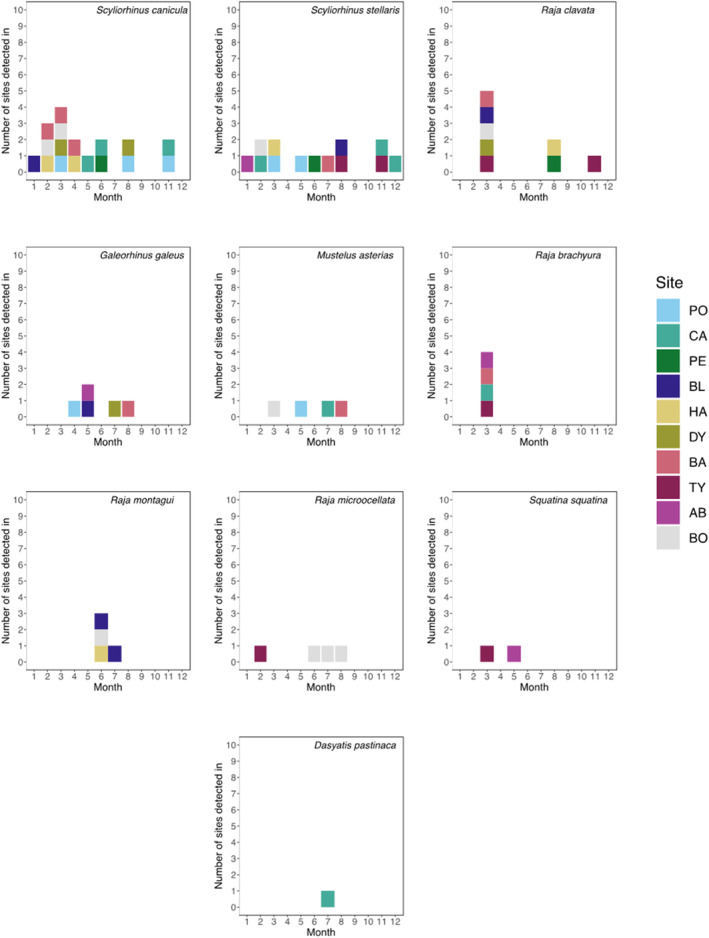
Number of detections for each elasmobranch species per month in PLAS SAC. Months are numbered according to the order January to December.

**FIGURE A3:**
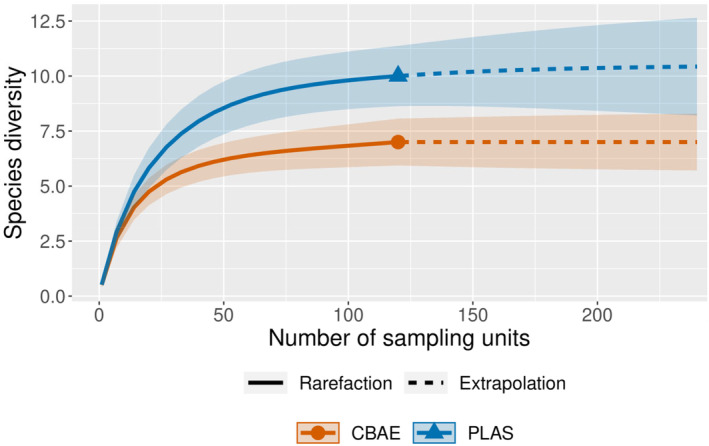
Species accumulation curves with 95% confidence intervals for elasmobranch species detection from eDNA in our surveys.

A total of 501 libraries were sequenced over 8 sequencing runs (120 samples, 120 negative controls from each SAC plus extraction and PCR blanks), resulting in 32,433,245 and 41,244,324 sequence reads from CBAE SAC and PLAS SAC respectively. At least one elasmobranch species was detected in 105 of the 240 water samples (43.8%). There were 123 discrete eDNA detections of an elasmobranch species and a total of eleven species were detected across the two SACs. In CBAE SAC, elasmobranch species were detected most often (14 eDNA detections) in June (Figure 2a) and in PLAS SAC, most detections (17) occurred in March and no elasmobranch species were detected in September and October (Figure 2b). In CBAE SAC, *Scyliorhinus canicula* (small‐spotted catshark) was detected most often, with 21 detections across seven stations; this was followed by *Raja microocellata* (Smalleyed ray), which was detected 13 times across seven stations (Figure 2a and Figure A1). In PLAS SAC, *S. canicula* was detected most often, with 17 detections across eight stations; this was followed by *Scyliorhinus stellaris* (nursehound) with 13 detections across nine stations (Figure 2b and Figure A2). Species accumulation curves suggest that a plateau was reached for elasmobranch species detection from eDNA at each SAC (Figure A3).

### 3.1 Carmarthen Bay and Estuaries SAC

A total of 2,193,035 paired sequences were taken forward for analysis after bioinformatic filtering and merging from the 2022/23 CBAE samples. After stringent removal of amplicon sequence variants (ASV) that were amplified in negative controls and after the removal of negative control samples, 100,801 sequence reads were retained of which elasmobranch species reads accounted for 1.12%. Seven elasmobranch species were identified (tope ( *Galeorhinus galeus* ), smallspotted catshark ( *Scyliorhinus canicula* ), nursehound ( *Scyliorhinus stellaris* ), starry smoothhound ( *Mustelus asterias* ), thornback ray ( *Raja clavata* ), small‐eyed ray ( *Raja microocellata* ) and undulate ray ( *Raja undulata* ). Figure 2a), and one further ASV was assigned to the genera *Raja* as it did not meet the confidence threshold for species level assignment, this record was not used in any subsequent analysis.

**FIGURE 3:**
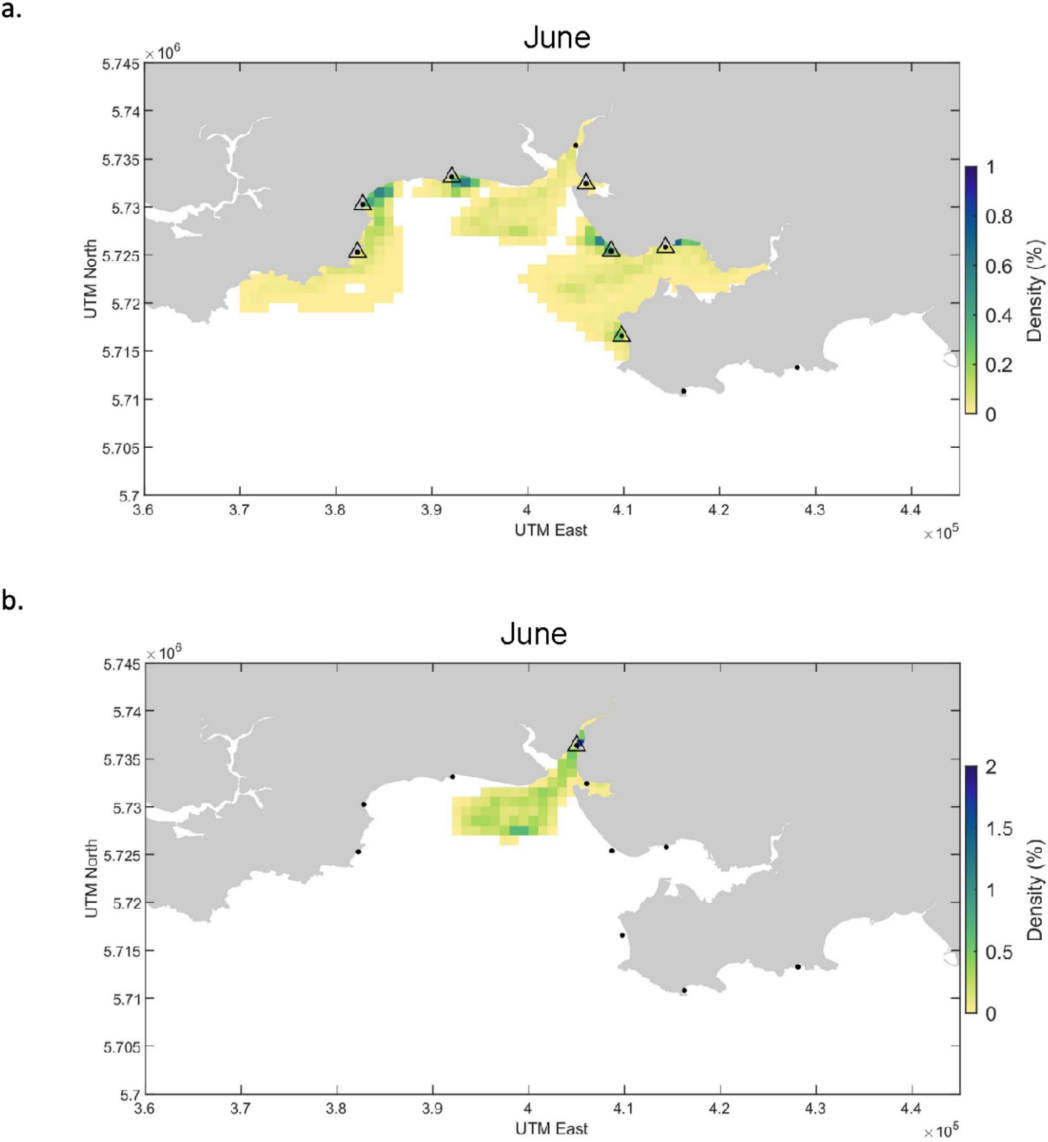
(a) Detections of *Mustelus asterias* in CBAE SAC (June 2022) and (b) detection of *Galeorhinus galeus* in CBAE SAC (June 2022). Black dots represent all sampling stations and triangles around a dot indicate an eDNA detection of the species at that station (in June 2022). The density score represents the percentage of particles present in each 1 km^2^ grid cell over the 3‐day backtracking simulation.

In June, *Mustelus asterias* was detected at seven of the ten stations and the backtracking simulations predicted that eDNA particles related to this detection could have been distributed throughout the bay within the boundaries of the SAC with maximum dispersal distances of 15 km over 3 days before reaching the sampling station (Figure 3a). The highest densities of simulated particles were estimated to be within a 1–2 km^2^ radius of the sampling stations (Figure 3a). The Critically Endangered *G. galeus* was detected at just one station and model estimates suggest that the eDNA particles may have originated from within the Three Rivers estuary, or from a maximum of 16 km further out into Carmarthen Bay whereby the eDNA was subsequently washed inshore to the sample location (Figure 3b).

**FIGURE A4:**
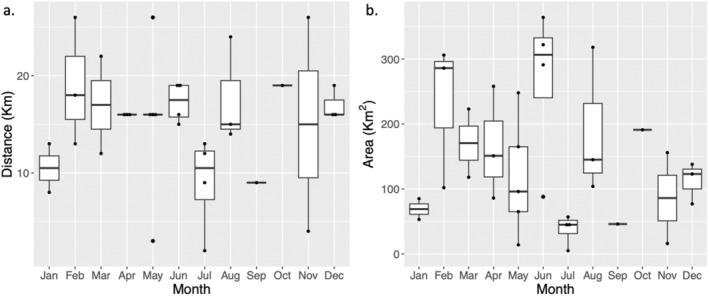
The maximum distances travelled (a) and surface area covered (b) by backtracked particles from each elasmobranch detection in CBAE SAC.

The mean of the maximum straight‐line distances of backtracked particle trajectories from each elasmobranch detection station during the simulation was ~15 km (standard deviation (sd) = 6; Figure A4a). The average surface area covered by backtracked particles from elasmobranch detections was 145 km^2^ (sd = 102) and the largest areal extent of particle locations in the backtracking simulations was 364 km^2^ (Figure A4b).

### 3.2 Pen Llŷn a'r Sarnau SAC

Analysis for the 2020/21 monthly surveys in PLAS comprised 5,946,334 paired sequences after bioinformatics filtering and merging, 1,273,051 were retained after removal of amplicon sequence variants (ASV) that were amplified in negative controls and the removal of negative control samples. Ten elasmobranch species were detected common stingray ( *Dasyatis pastinaca* ), tope ( *Galeorhinus galeus* ), smallspotted catshark ( *Scyliorhinus canicula* ), nursehound ( *Scyliorhinus stellaris* ), angelshark ( *Squatina squatina* ), starry smoothhound ( *Mustelus asterias* ), thornback ray ( *Raja clavata* ), small‐eyed ray ( *Raja microocellata* ), spotted ray ( *Raja montagui* ) and blonde ray ( *Raja brachyura* ) (Figure 2b), accounting for 5.28% of the retained reads.

**FIGURE 4:**
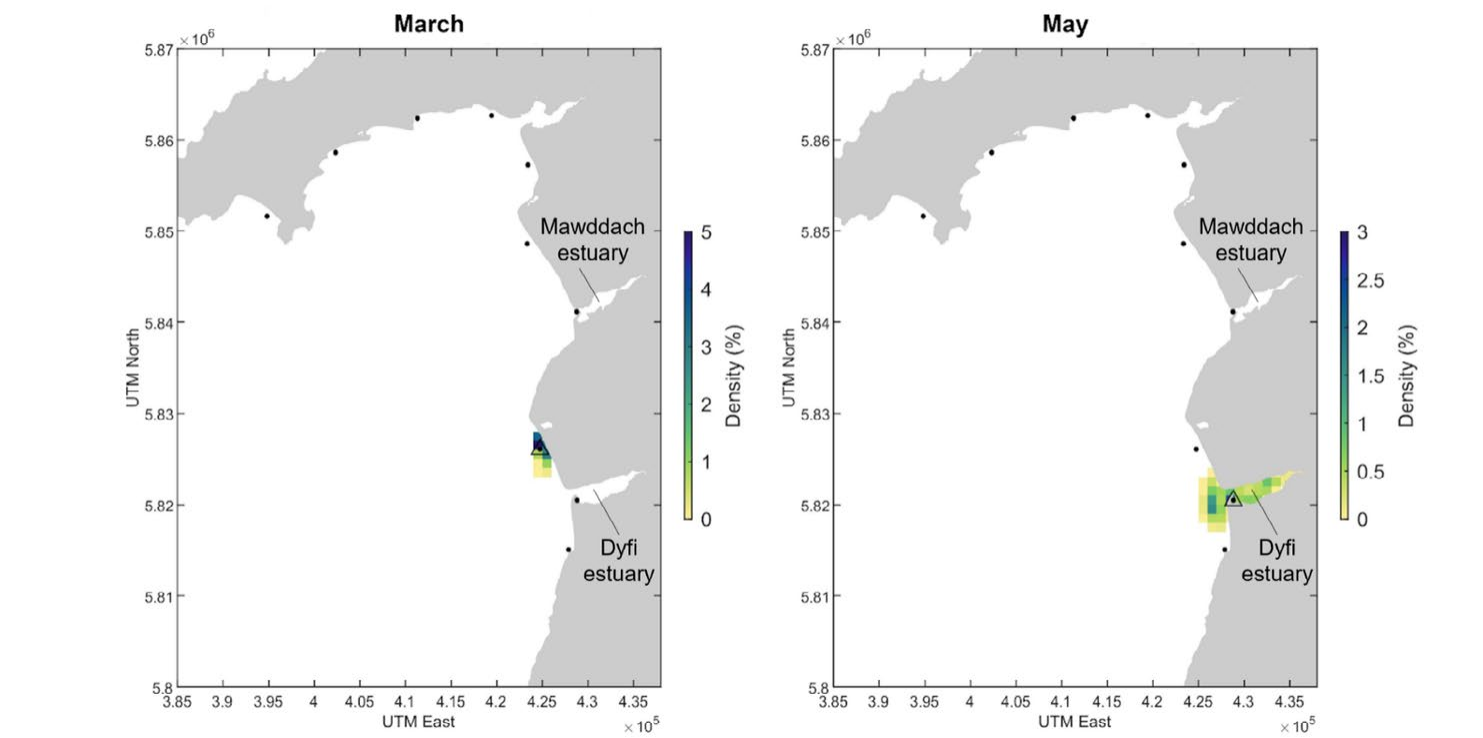
Detections of *Squatina squatina* in PLAS SAC in March and May 2021. Black dots represent sampling stations and triangles around a dot indicate an eDNA detection of the species at that station. The density score represents the percentage of particles present in each 1 km^2^ grid cell at every 5‐min time interval in the 3‐day backtracking simulation. The areal extent of the simulated particle dispersal covers 10 km^2^ (March) and 42 km^2^ (May).

**FIGURE 5:**
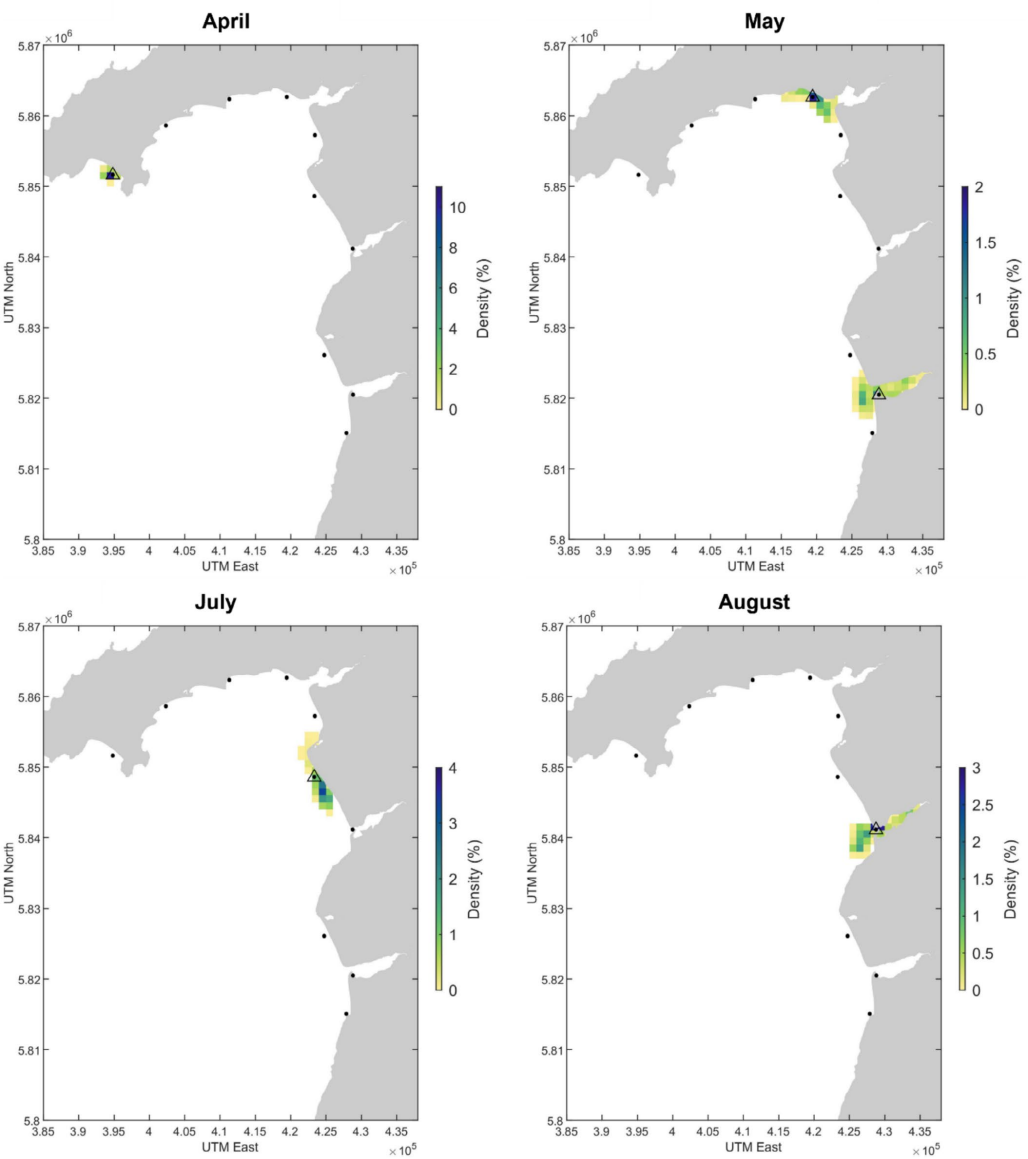
Detections of *G. galeus* in PLAS SAC in April, May, July and August 2021. Black dots represent sampling stations and triangles around a dot indicate an eDNA detection of the species at that station. The density score represents the percentage of particles present in each 1 km^2^ grid cell over the 3‐day simulation period. The areal extent of the simulated particle dispersal covers 7 km^2^ (April), 65 km^2^ (May) (across two detections), 27 km^2^ (July) and 34 km^2^ (August).

The Critically Endangered *Squatina squatina* (angelshark) was detected in March and in May, and model backtracking predicts that the eDNA could have travelled a maximum of 3 km in March and 9 km in May to reach the sampling stations. In May, the highest density of backtracked particles at 3 days before sampling is near to the shore and within the Dyfi estuary in May (Figure 4). The Critically Endangered *G. galeus* was detected across 4 months in the PLAS SAC, the model backtracking indicated that the eDNA may have originated from the Dyfi and Mawddach estuaries and particles are estimated to have been transported a maximum of 2 km (April), 9 km (May), 6 km (July) and 6 km (August) and remained inshore (Figure 5).

**FIGURE A5:**
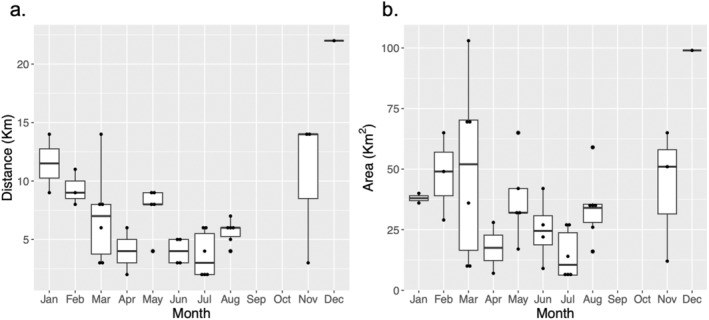
The maximum distances travelled (a) and surface area covered (b) by backtracked particles from each elasmobranch detection in PLAS SAC.

The mean of the maximum straight‐line distances of the backtracked particle trajectories from each eDNA elasmobranch detection was ~7 km (sd = 4), the greatest estimated distances covered were in the winter months (Figure A5a) and the average areal extent covered by backtracked particles from elasmobranch detections was 36.5 km^2^ (sd = 24.6) (Figure A5b).

### 3.3 Connectivity Between Sample Stations

**FIGURE 6:**
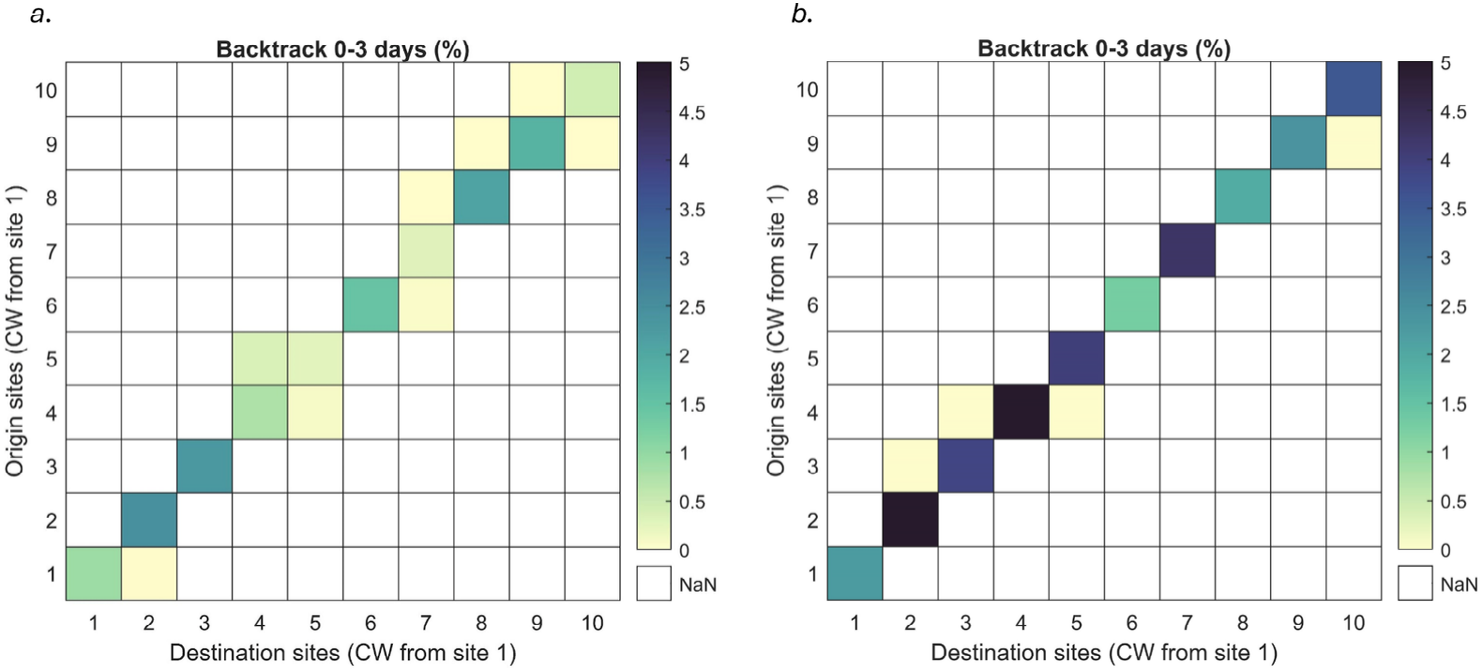
Connectivity heatmaps showing the backtrack particle mixing between stations for (a) CBAE SAC and (b) PLAS SAC for 3 days at 1 km^2^ resolution. The colour scales indicate the number of particles as a percentage of the total particles released from each of the 10 stations. Stations are numbers 1–10 are arranged in a clockwise (CW) direction as follows: (a) TS, SA, PE, FE, SI, PB, BU, LL, PO and CA and (b) PO, CA, PE, BL, HA, DY, BA, TY, AB, BO (see Figure 1).

By recording the particle locations throughout the three‐day period before the sampling was conducted, it was possible to estimate whether there was duplication within the eDNA results (i.e., whether water could have flowed through more than one station during those 3 days). Particle tracking in the two SACs from the hydrodynamic models showed that there was potentially some ‘mixing’ between stations at both SACs. In CBAE SAC, there was potential for mixing between six pairs of stations in the three‐day time period considered here, with the only station 9 (PO) potentially connected to two others (the remainder was for connectivity to only one station) (Figure 6). In PLAS SAC, there was less connectivity between sampling stations with four pairs of stations potentially connected, only one station (4, BL) was potentially connected to more than one other station. Overall, there was less dispersal from the sampling stations in PLAS SAC than in CBAE SAC, with higher densities of particles remaining within the 1 km radius of release considered here (i.e., darker blue colours in Figure 6b. than in Figure 6a).

## 4 Discussion

In this study, we demonstrate the use of eDNA metabarcoding for detecting elasmobranch species in highly turbid, temperate water. As this environment typically presents a challenge for monitoring elasmobranch species using conventional survey techniques (such as BRUVS and UVC), eDNA methods in conjunction with hydrodynamic modelling and particle tracking, present an opportunity to develop our understanding of the spatial and temporal distribution of these species. Our results include the detection of Critically Endangered species, show that the eDNA detection of elasmobranch species was temporally variable and the average maximum distance travelled by the eDNA was 15 km (CBAE SAC) and 7 km (PLAS SAC) from the sampling stations. This strengthens support for the use of eDNA for marine species monitoring and confirms that eDNA methods are relevant for spatial conservation and associated species feature management in Welsh waters and SACs.

Environmental DNA is known to be ephemeral in the marine ecosystem and there is a complex relationship between eDNA state and environmental factors when considering eDNA persistence (Jo and Minamoto 2021). Numerous studies have attempted to determine the decay rates of eDNA in water with calculated half‐lives ranging from 6.9 h in 22°C water (Sassoubre et al. 2016) to 63 h in 4°C water (Weltz et al. 2017). Collins et al. (2018) estimated the eDNA persistence time in a similar environment to that of the samples in this study was around 48 h. To account for this and to envelop other limitations within the modelling techniques (time steps, grid resolution etc.), we performed particle backtracking to simulate particle movement during the 3 days (72 h) preceding each specific sampling time. This modelling provides us with an estimate of where eDNA particles may have been advected from within coastal waters, thus indicating the potential areal extent within which the detected species may have been within the 3 days prior to water sampling. It is possible that eDNA detected for a given species originated from multiple individuals however, we are not able to differentiate individuals from eDNA metabarcoding analyses. The particle tracking model is not linked to an individual and therefore the backtracking simulations may represent the estimated distribution of multiple individuals from a detected species. We also assumed that eDNA behaves identically for each species and as such each backtracking simulation is not targeted to a given species. It is possible that eDNA derived from different species behaves differently in the water (e.g. Andruszkiewicz Allan et al. 2021), but significant research effort will be needed to determine and parameterise this for elasmobranch species. As highlymobile taxa, individuals from the detected species will have been constantly on the move during the sampling period. Our results are aimed at representing a snapshot of where eDNA could have been shed from, and therefore provide estimates of the locations of any number of individuals from the detected species in the 3 days prior to sampling. Using these methods to assimilate knowledge of the diversity and likely distribution of elasmobranch species attained in this study may allow sites to be managed accordingly for their conservation in the future.

There is evidence that the majority of eDNA detections for fish are from samples taken within 30 m of the eDNA's origin (Murakami et al. 2019), suggesting that eDNA provides a snapshot of the organisms present at the time of sampling. Our results suggest that within 3 days, eDNA particles can travel distances in the tens of kilometres from its origin before detection and can be distributed over an area in the tens or hundreds of km^2^. The estimated detection areal extents allow us to make predictions of species distributions and highlight the lens through which eDNA detections should be considered in marine studies. The modelling results indicate that sampling stations in CBAE were largely independent of one another and that eDNA sampled at a given sampling station is most likely to have originated from around that station or from one of the neighbouring stations. The water in PLAS SAC showed limited connectivity between sampling stations, suggesting that there was little chance of detections at multiple stations originating from the same source. Our model did not account for differences in eDNA detection rates across the year driven by water temperatures or for differences in the transport of eDNA particles in different states (i.e., extracellular, cellular, adsorbed). Attempts should be made to quantify the impacts of these differences in marine systems so that they can be properly accounted for in eDNA particle tracking models.

There are 27 elasmobranch species known to inhabit the coastal waters around Wales (SIARC, 2022). From a total of 240 monthly samples taken from two SACs, we detected eleven of these species. There was at least one elasmobranch species detected in 105 samples (43.8%), with a total of 123 detections. The species accumulation curves suggest that species detection had plateaued at each site, showing that eDNA methods are suitable for detecting elasmobranch species richness in turbid systems that present challenges for more established monitoring methods, such as BRUVS Further, the number of species detected here is comparable to the numbers recovered by Liu et al. (2022) in their study in the English Channel. However, despite using elasmobranch‐targeted primer sequences in the metabarcoding, elasmobranch reads only accounted for a low percentage of reads, highlighting the need for continued improvement of elasmobranch eDNA assays to ensure that rare sequences and species are not overlooked in analyses. We took a negative control for each water sample and used these to stringently remove any possible contamination in our samples thus resulting in significantly parred down data for analysis that represents the true structure of DNA in the samples. Significant sequencing effort was effectively wasted on sequencing human‐derived eDNA and this could have resulted in lower amplification of low abundance sequences from rare species in the samples. The use of suitable blocking primers to prevent this should be further explored for studies targeting elasmobranch eDNA. The species we did detect were predominantly benthopelagic, reflecting the shallow, intertidal waters sampled in this study. Sampling across wider habitats, further offshore and at different depths would be required to capture the full diversity of elasmobranchs in Wales. The majority of elasmobranch species detected were shared across PLAS and CBAE samples. *S. canicula* and *S. stellaris* are known to be common off the Welsh Coast and were detected in all regions. *R. clavata* was also detected in all the regions despite documented decreases in population in the PLAS region (Whittamore and Mccarthy 2005).

The detection of *S. squatina* in PLAS SAC at an estuarine station suggests that the region could be important for this Critically Endangered species. Particle backtracking suggests that the eDNA is likely to have originated near the site of detection (< 9 km). Furthermore, this area does represent a location with a high number of catch records for the species historically (Barker et al. 2022). As the species was only detected in March and May, future surveys should focus on this region in the spring to investigate whether this site represents an area of interest for the species. There was no detection of *S. squatina* in CBAE SAC despite it being a historical hotspot for the species (Barker et al. 2022). Significantly lower reads were retained as ‘true’ detections in the CBAE SAC samples than PLAS SAC samples (100,801 vs. 1,27,051), this was largely due to a vast number of sequencing reads being attributed to *Homo sapiens* in the actual and control samples which were subsequently removed from the dataset, therefore it may be the case that rare sequences in the sample were swamped out by the high abundance of human DNA and impacted the ability to detect eDNA from rare species in the samples. If PLAS SAC remains a stronghold for *S. squatina* in Welsh waters and in the wider Irish Sea, it is important that focused research and fisher engagement continues in the area to ensure the species can re‐establish its historical range.

These results have provided us with information on the spatial and temporal distribution of elasmobranch species in PLAS and CBAE SACs, demonstrating the effectiveness of eDNA methods when used in temperate and turbid systems. The turbidity of the water did present a challenge to the filtration method and multiple filters were often required to filter 2 L of water. Pre‐filtering methods should be considered for future sampling events in these locations and the use of passive samplers could reduce the need for filtering altogether (Maiello et al. 2022). However, studies are required to ensure that the use of these methods would not reduce the detection rates of elasmobranch eDNA in this system. Future research should also consider investigating how eDNA decay rates relate to water turbidity and if this can be parameterised for modelling. Whilst there was no indication of sample inhibition during the amplification process, it is possible that eDNA taken from highly‐turbid water systems could contain substances that would inhibit PCR. DNA clean up steps should be built into eDNA workflows for these systems as a matter of course to ensure that high integrity and reliable data is produced when used for biodiversity monitoring.

Hydrodynamic modelling and particle tracking studies remain underutilised tools in eDNA studies yet have the potential to increase the confidence that is attributed to detections and therefore elevate eDNA methods to become established in biodiversity monitoring for spatial management. As there is little information about elasmobranch species distributions in the study region, these findings can now be used to target species for more in‐depth ecological research so that we can gain a deeper understanding of the ecology of elasmobranchs off the coast of Wales. This will allow us to ensure that elasmobranch species and their relationship with SAC habitat features is better understood which may inform fisheries management and other activities within the SACs. Ultimately allowing populations to recover to historical levels and ensuring that elasmobranchs can continue to provide essential ecosystem services along the UK coast.

## Author Contributions

**Nick Dunn:** conceptualization (lead), data curation (equal), formal analysis (lead), investigation (lead), methodology (lead), visualization (lead), writing – original draft (lead), writing – review and editing (equal). **Sophie Ward:** data curation (equal), formal analysis (lead), software (lead), visualization (lead), writing – original draft (supporting), writing – review and editing (equal). **Jake Davies:** conceptualization (supporting), funding acquisition (supporting), methodology (supporting), writing – review and editing (supporting). **Sarah Davies:** conceptualization (supporting), funding acquisition (supporting), methodology (supporting), writing – review and editing (supporting). **Kevin Hopkins:** data curation (supporting), methodology (supporting), resources (supporting), software (supporting), writing – review and editing (supporting). **Isabelle Apetroaie:** methodology (supporting), writing – review and editing (supporting). **Jake Williams:** data curation (supporting), formal analysis (supporting), software (lead), writing – review and editing (supporting). **Ben Wray:** conceptualization (supporting), funding acquisition (supporting), project administration (supporting), writing – review and editing (supporting). **Joanna Barker:** conceptualization (lead), funding acquisition (lead), methodology (supporting), project administration (lead), writing – review and editing (supporting). **Peter Robins:** conceptualization (supporting), data curation (supporting), software (supporting), supervision (supporting), writing – review and editing (supporting). **David Curnick:** conceptualization (supporting), funding acquisition (supporting), project administration (supporting), supervision (lead), writing – original draft (supporting), writing – review and editing (equal).

## Conflicts of Interest

The authors declare no conflicts of interest.

## Data Availability

Raw sequence reads are available from Dryad (DOI: https://doi.org/10.5061/dryad.m0cfxppcx). Bioinformatics scripts are available here: J‐Cos/SimpleMetaPipeline: 2.0.2. Particle tracking and analysis scripts are available here: https://zenodo.org/records/14391532.

## Appendix A

**TABLE A1:** River flow rate data and the corresponding station number used in the modelling.

River	Mean annual flow rate (m^3^/s)	Station number
PLAS SAC		
Rheidol	8.51	63,002
Dyfi	23.31	64,001
Dysynni	4.44	64,002
Mawddach	3.97	64,010
Glaslyn	5.84	65,001
CBAE SAC		
Loughor at Tir‐Y‐Dail	2	4131
Tywi at Capel Dewi	40	4139
Gwili at Glangwyli	5	4199
Dewi Fawr at Glaslyn Ford	1	4096
Taf at Clog Y Fran	7	4089
